# Household Hints and Recipes

**Published:** 1875-07

**Authors:** 


### Household Hints & Recipes.

PASTE THAT ADHERES TO TIN.—Add to ordinary paste some honey or glycerine. Muriatic or tartaric acids are also used for the purpose.

---

TO BLEACH SPONGES:—To bleach sponge, wash first in weak muriatic acid, then in cold water; soak in weak sulphuric acid, wash in water again, and finally rinse in rose-water.

---

TO IMITATE GROUND GLASS:—A ready way of imitating ground glass is by dissolving Epsom salts in ale (don't use this as a beverage) and applying with a brush; as it dries it crystallizes.

---

STARCH ENAMEL.—Spermaceti and white wax are sometimes added to starch with the intention of giving a shining appearance to the clothes on which it is used. Stearine is also frequently added for the same purpose.

---

WARTS.—Dr. Guttceit recommends rubbing warts, night and morning, with a moistened piece of muriate of ammonia. They soften and dwindle away, leaving no such white mark as follows their dispersion with lunar caustic.

---

CEMENT TO UNITE BRASS AND WOOD.—The *English Mechanic* says that the best cement for this purpose is a glue composed of best gelatine 1 part, glacial acetic acid 1 part. Soak the gelatine in cold water until it has swollen up and become quite soft. Throw away the water and dissolve the gelatine in the acetic acid, applying gentle heat if necessary.

---

AN AMUSING EXPERIMENT.—The *English Mechanic* says: "Cut (if you dare) four small, white feather-points from your wife's best bonnet, and insert them into two small pith-balls, in imitation of wings. Fasten these butterflies to two fibres of raw silk of about a foot in length, and suspend the whole from the ceiling by means of another fibre. Excite the chimney of a moderator lamp with a silk handkerchief, give the glass to one of your young friends, and request him to persuade the butterflies to enter the tube. His ineffectual attempts to capture the butterflies will create much merriment. Breathe momentarily into the tube, or remove the electricity by some other unobserved method, and, of course, the feat is easily accomplished, while the astonishment of the beholders is intensified.

LINENS, TO WASH.—A tablespoonful of black pepper put in the first water in which gray and buff linens are washed, will keep them from spotting. It will also keep the colors of black or colored cambrics or muslins from running, and does not harden the water. A little gum arabic imparts a gloss to ordinary starch.—*Bazar.*

---

COLOGNE WATER.—A mixture of oils is made as follows: Oil of neroli 2 p., oil of rosemary 1 p., oil of lemon 3 p., oil of bergamot 1 p., and oil of orange 3 p. One kilogramme of this mixture is dissolved in 60 litres of alcohol (85 to 90 per cent.,) the solution heated to 60° C. (140° F.) and subsequently filtered. The heating effects the blending of the perfumes in a short time, which otherwise takes place only after several months.—*Jour. Pharm.—Pharm. Centr. Halle.*

---

SWEATING FEET.—This disagreeable affliction seems to depend either upon abnormal perspiration, want of cleanliness, or the wearing of non-ventilating shoes and stockings. The remedies in the two latter cases are obvious, but in the first case it is well-nigh incurable, although the disagreeable effects may be lessened. Cooley recommends frequent washing, with the addition to the water of a teaspoonful of chloride of lime, or double that quantity of common salt. A cup of strong vinegar added to the wash-water also has a good effect.

---

CABBAGE AND HAM SALAD.—The following recipe is from the *Country Gentleman:* Take two small heads of cabbage, well washed, and chop them quite fine; slice off a dozen or more thin slices of tender boiled ham. Mix the two together in a salad bowl. Make a dressing of two raw eggs, mixing the yolks with half a teespoonful of mustard, stirred up in boiling water; then add three tablespoonfuls of sour cream just skimmed from the pan, or one small teacupful of salad oil, poured in very slowly, as directed for mayonnaise. Stir for ten minutes, adding a little salt and pepper. Beat the whites of the eggs to a froth, and add to it also four tablespoonfuls of vinegar. This makes a delicious side dish, or a course at the dinner-table, and the housewife will find it a toothsome substitute for a hot dinner when the mercury mounts high up among the nineties, and there is ironing or washing to attend to. Chopped cold boiled potatoes can also be added, and the dish can be prepared out of cold boiled corned beef chopped fine, or from cold roast veal, beef or mutton. Lettuce can be substituted for cabbage if preferred, and the mustard can be left out.

GUTTA-PERCHA CEMENT FOR FASTENING LEATHER.—Dissolve some crude gutta-percha in chloroform or bisulphide of carbon, in quantity sufficient to give the liquid a honey-like consistence. Apply this solution to the surfaces to be united, allow it to dry (which takes only a few moments,) then soften by exposure to the heat of a gas flame and unite the parts firmly.

This cement can be used for patching shoes, and uniting leather generally, with excellent effect.

---

HOW TO TEST THE QUALITY OF A PEAR.—In the *Revue de l'Horticulture Belge*, M. Buchetet gives a test for ascertaining the quality of a pear. If, says he, you can write on it easily with pen and ink, it is a good pear. Other pears, whose rind does not take the ink so well, are inferior in flavor. It would be curious if this rule did not, like most rules, have its exceptions. So good an authority whould hardly have given it without having tested its application to the varieties of the fruit that are most common in Belgium. No doubt some of our readers will be interested to see how it works on this side of the Atlantic.

---

MODE OF DISCOVERING WHETHER RED WINES ARE ARTIFICIALLY COLORED OR NOT.—M. de Cherville, in one of his clever agricultural articles in *Le Temps*, gives the following useful hints for deciding the above:—"Pour into a glass a small quantity of the liquid which you wish to test, and dissolve a bit of potash in it. If no sediment forms, and if the wine assumes a greenish hue, it has not been artificially colored; if a violet sediment forms, the wine has been colored by elder or mulberries; if the sediment is red, it has been colored with beet root, or Pernambuco wood; if violet-red, with logwood; if yellow, with phytolac berries; if violet-blue, with privet berries, and if pale violet, with sunflower."

---

SINGULAR MATHEMATICAL FACT.—Any number of figures you may wish to multiply by 5 will give the same result if divided by 2—a much quicker operation; but you must remember to annex a cipher to the answer, whenever there is no remainder, and when there is a remainder, whatever it may be, annex 5 to the answer. Multiply 464 by 5 and the answer will be 2,320; dividing the same number by 2 and you have 232, and as there is no remainder, you add a cipher. Now take 357 and multiply by 5; there is 1,785. Divide the same number by 2, and you have 187 and a remainder; you therefore place a 5 at the end of the line and the result is again 1,785.

A MATCH UNDER THE MICROSCOPE.—A correspondent of the *Scientific American* writes as follows: "Those who are fond of investigations with the microscope will find a beautiful object in the head of a parlor match. Strike the match, and blow it out as soon as the head has fused sufficiently to cause protuberances to form on it; on the part of the head which took fire first, will be found a white, spongy formation, which, under the microscope and with a bright light upon it, has the appearance of diamonds, crystals, snow, frost, ice, silver and jet, no two matches giving the same combination or arrangement."

---

DETECTION OF BEEF FAT OR LARD IN BUTTER.—Mr. Stoddart gives the following method of distinguishing betwecn butter and other fats of animal origin. A quantity, say fifty grains, of butter, is put into an ounce bottle, half filled with ether, and the mixture is well agitated. If the butter be genuine, perfect solution of the fatty matter will take place, and salt and water will be separated, together with curd, which is occasionally present to the extent of eight or nine per cent. The salt and water may be readily recognized, and the curd may be proved such by heating a small portion on a slip of glass, when it will dry and fall to powder. If beef fat or lard be present, they will not dissolve in the ether, but fall to the bottom of the solution; by the application of heat, as in the case of curd, the fatty character of these substances is at once shown by their liquefaction.

---

NICKEL PLATING.—The following is a simple and easy method for nickel plating, highly recommended by Prof. Stolba: "Into the plating vessel which may be of porcelain, but preferably should be of copper, is placed a concentrated solution of chloride of zinc, which is then diluted with one to two volumes of water, and heated to boiling. If any precipitate is formed it is redissolved by the addition of a few drops of hydrochloric acid. As much powdered zinc as can be placed upon the point of a knife is thrown into the solution, and the inside of the vessel becomes covered with a coating of zinc. Either chloride or sulphate of nickel is then added until the liquid is distinctly green, and the articles to be plated, previously thoroughly cleaned, are introduced together with some zinc fragments. The boiling is continued fifteen minutes, when the nickel coating is finished. The articles are to be well washed with water and then rubbed with chalk. If a thicker layer of deposited metal is desired, the operation can be repeated.

INDIA-RUBBER CEMENT.—

Gutta-percha, 3 parts.
Pure India-rubber, 1 part.
Bisulphide of carbon, 8 parts.

Mix in a close vessel, and dissolve by the aid of a gentle heat, applied by the water bath. The gutta-percha and rubber should be cut into small pieces previous to solution.

The cement must be gently heated before applying to the articles to be mended.

By writing to your wholesale drug or paint house you can probably find out where you may procure pure rubber or gutta-percha.

---

PASTE.—A brilliant and adhesive paste, adaped to the uses of manufacturers of fancy articles, painters, etc., may be made by dissolving caseine, precipitated from milk by acetic acid and washed with pure water, in a saturated solution of borax. Apropos of paste, an English writer says; "Mix good clean flour with cold water into a paste, well blended together, then add boiling water, stirring well until it is of such a consistency that it can be easily and smoothly spread with a brush; add to this a spoonful or two of brown sugar, a little corrosive sublimate, and about half a dozen drops of oil of lavender, and you will have a paste fit to fasten the teeth in a saw."

---

COLORLESS VARNISH.—Dissolve 2½ ounces of shellac in a pint of rectified alcohol; boil for a few minutes with 5 ounces of well-burned and recently-heated animal charcoal. A small portion of the solution should then be filtered, and if not colorless, more charcoal must be added. When all color is removed press the liquor through a piece of silk, and afterwards filter through fine blotting-paper. This kind of varnish should be used in a room heated up to at least 60° F., perfectly free from dust. It dries in a few minutes, and is not liable afterwards to chill or bloom. It is particularly applicable to drawings and prints that have been sized, and may be used for gilding.

---

BASKET PLANTS.—Basket plants often suffer from too much or too little water. If too much, they get yellow and drop off. As a rule a basket in a warm room should be taken down once a week; soaked in a bucket of water; then drained and hung up again. Every day during the rest of the week a little water may be given the plants, and something put under to catch the drip. Some baskets have no provision for the escape of moisture. These are dangerous. Still some people manage to watch closely, and do well with them. Fern cases do best when given a little sun; for, though ferns are supposed to grow naturally in shady spots, it is because there generally is a more humid atmosphere. If they can get this moisture they rather like the light.—*Gardener's Monthly.*

---

TO KEEP EGGS OVER WINTER.—The *Farmer's Advocate*, London, Ontario, recently offered a prize for the best method of keeping eggs over winter.

The first receipt given below took the prize:

Whatever excludes the air prevents the decay of the egg. What I have found to be the most successful way of doing so, is to place a small quantity of salt butter in the palm of the hand and turn the egg round in it, so that every pore of the egg-shell is closed; then dry a sufficient quantity of bran in an old oven (be sure you have the bran well dried, or it will rust). Then pack them with the small ends down, a layer of bran and another of eggs, until your box is full; then place in a cool, dry place. If done when new laid, they will retain the sweet milk and curd of a new-laid egg for at least eight or ten months. Any oil will do, but salt butter never becomes rancid, and a very small quantity of butter will do a very large quantity of eggs. To ensure freshness I rub them when gathered in from the nests; then pack when there is sufficient quantity.

---

TO DISTINGUISH WHOLESOME FROM UNWHOLESOME MEAT.—The London *Sanitary Record* says: "The characteristics of sound, wholesome meat are that it is firm and elastic to the touch, and when pressed by the hand it does not retain any indentation. It should have little or no odor. The lean should have a 'marbled' appearance, and should not turn watery or become wet after standing a day or two. Avoid meat which is a pale pink color, which is an indication of disease, or of a deep purple color, which is a sign that the animal has either died a natural death or suffered from acute fever. A little practice and observation will readily enable you to discriminate wholesome from unwholesome meat. It would be as well not to make your purchases by night; the characteristics of good meat can be best seen by daylight. So well, indeed, was this fact understood by the city authorities of old, that the 'Liber Albus' contains an exactment 'that butchers shall close their shops before candle-light, and shall not sell meat by the light of candle.'"

